# Substrate Cooperativity in Marine Luciferases

**DOI:** 10.1371/journal.pone.0040099

**Published:** 2012-06-29

**Authors:** George Tzertzinis, Ezra Schildkraut, Ira Schildkraut

**Affiliations:** 1 New England Biolabs, Inc., Ipswich, Massachusetts, United States of America; 2 CerroSci LLC, Ipswich, Massachusetts, United States of America; U. Kentucky, United States of America

## Abstract

Marine luciferases are increasingly used as reporters to study gene regulation. These luciferases have utility in bioluminescent assay development, although little has been reported on their catalytic properties in response to substrate concentration. Here, we report that the two marine luciferases from the copepods, Gaussia princeps (GLuc) and Metridia longa (MLuc) were found, surprisingly, to produce light in a cooperative manner with respect to their luciferin substrate concentration; as the substrate concentration was decreased 10 fold the rate of light production decreased 1000 fold. This positive cooperative effect is likely a result of allostery between the two proposed catalytic domains found in Gaussia and Metridia. In contrast, the marine luciferases from Renilla reniformis (RLuc) and Cypridina noctiluca (CLuc) demonstrate a linear relationship between the concentration of their respective luciferin and the rate of light produced. The consequences of these enzyme responses are discussed.

## Introduction

**Figure 1:**
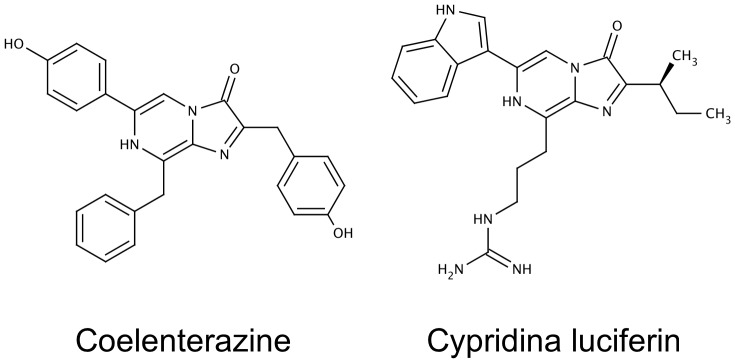
Chemical structure of Coelenterazine and Cypridina luciferin.

Luciferases, the enzymes responsible for the bioluminescence reaction, are present in multiple animal phyla and bacteria. The luciferases oxidize luciferins to produce light and the chemical nature of the luciferins can vary widely. Perhaps the best known are the ATP-dependent beetle luciferases that catalyze the oxidation of firefly luciferin in a photochemical reaction that has been widely used for the detection of low levels of ATP. In contrast, most marine luciferases do not use ATP, requiring only their luciferin and molecular oxygen as substrates with oxyluciferin, CO_2_ and light as products. Different species use different luciferins: coelenterazine is the cognate luciferin for Renilla, Gaussia and Metridia luciferases (RLuc, GLuc and MLuc, respectively), while cypridina luciferin is the cognate luciferin for Cypridina luciferase (CLuc) (Figure 1). The two chemically related luciferins share the common chromophore, imidazopyrazinone.

**Figure 2:**
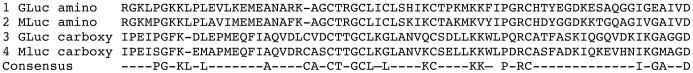
Alignment of both the amino terminal and carboxyl terminal halves of GLuc and MLuc. 1) GLuc amino terminal half. 2) Mluc amino terminal half. 3) GLuc carboxyl terminal half. 4) MLuc carboxyl terminal half.

Some of the marine luciferase proteins have been extensively studied and mutated in order to improve their yield and bioluminescent properties [1]–[8]. The three dimensional structure of RLuc has been solved. It is a 37 kDa monomer and it contains a single substrate binding site [9]. CLuc is a 62 kDa protein [10]. GLuc and MLuc are 18.5 kDa and 22 kDa respectively with similar amino acid sequence and both contain a duplication of a proposed catalytic domain [1], [2] Figure 2. RLuc and CLuc sequences are neither similar to each other nor to GLuc or MLuc.

In the past twenty years, the genes of *Renilla* [11], and more recently *Gaussia* [12], [13], *Metridia* [14] and *Cypridina* [10] luciferases have been used as expression reporters for determining how particular genes are regulated by placing the luciferase gene downstream of particular regulatory sequences. Typical reporter assays measure the amount of luciferase protein that has been produced by measuring its activity in relative light units (RLU) from cell extracts or conditioned media by adding nearly saturating luciferin concentrations. Furthermore bioluminescent imaging of whole animals with marine luciferases has been used to identify tumors or tissue specific reporter gene expression [15]. Despite extensive structural and molecular biology characterizations, no classical kinetic characterization of these enzymes has been reported.

The range of sensitivity for detection of these luciferases spans many orders of magnitude. At a fixed high substrate concentration, very low levels of luciferase can be detected. In contrast here, it was of interest to determine whether the presence of very low levels of substrate (picomolar) could be detected in the presence of a fixed amount of luciferase. In this study we compared the kinetic properties of several marine luciferases in relation to their luciferin concentration, in an effort to identify a luciferase enzyme that offers the highest sensitivity in such an assay. Similar bioluminescent assays utilizing marine luciferases have been performed with RLuc in linked reactions to measure the concentration of 3′–5′ adenosine diphosphate (PAP) [16], [17]. This assay requires extreme sensitivity, and it was anticipated that luciferases having a higher rate of turnover than RLuc [18] e.g. GLuc [5] would perform substantially better for the detection of PAP.

## Results

**Figure 3:**
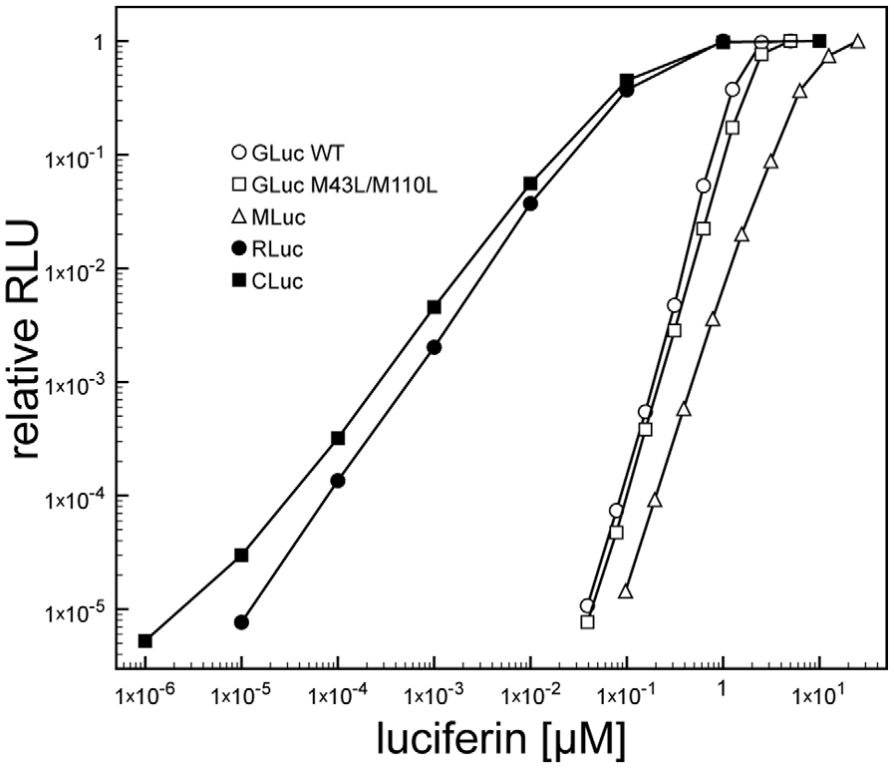
Activity of luciferases at various concentrations of luciferins. Plot of luciferase activity where a fixed amount of each luciferase was mixed with varying amounts of its corresponding luciferin.

In examining the detection limit of the marine luciferases for their luciferins, a fixed amount of luciferase was incubated with varying concentrations of its corresponding luciferin and the light generated was measured. Figure 3 shows the light released during the first 10 seconds of the reaction from GLuc, GLuc (M43L/M110L), RLuc, MLuc and CLuc luciferases over a large range of luciferin concentrations. The quantity of light generated in 10 seconds with RLuc was nearly linear with respect to the substrate over a 5 order of magnitude range of concentration: for every 10 fold decrease in coelenterazine concentration the light decreased about 10 fold. Likewise the rate of light generation by CLuc was linear with respect to the concentration of its substrate, cypridina luciferin. However GLuc, MLuc and the variant GLuc M43L/M110L of Welsh et al. [5] displayed a non-linear response to substrate concentration; as the substrate concentration decreased 10 fold the light generated decreased about 1000 fold. The same non-linear response was observed with GLuc that was expressed and secreted from CHO cells (data not shown) indicating that this property is not a result of how the protein was produced and processed in E. coli. Additionally, whether these reactions were performed in 20 mM Tris-HCl pH 7.5, 100 mM NaCl buffer as shown, or in a commercial assay buffer containing a signal stabilizer (Biolux GLuc Flex Assay Buffer), the same effect was observed (data not shown). Therefore it appears the luciferase reaction catalyzed by CLuc and RLuc are apparent first order with respect to their luciferin, while GLuc and Mluc are higher order with respect to their luciferin.

**Figure 4:**
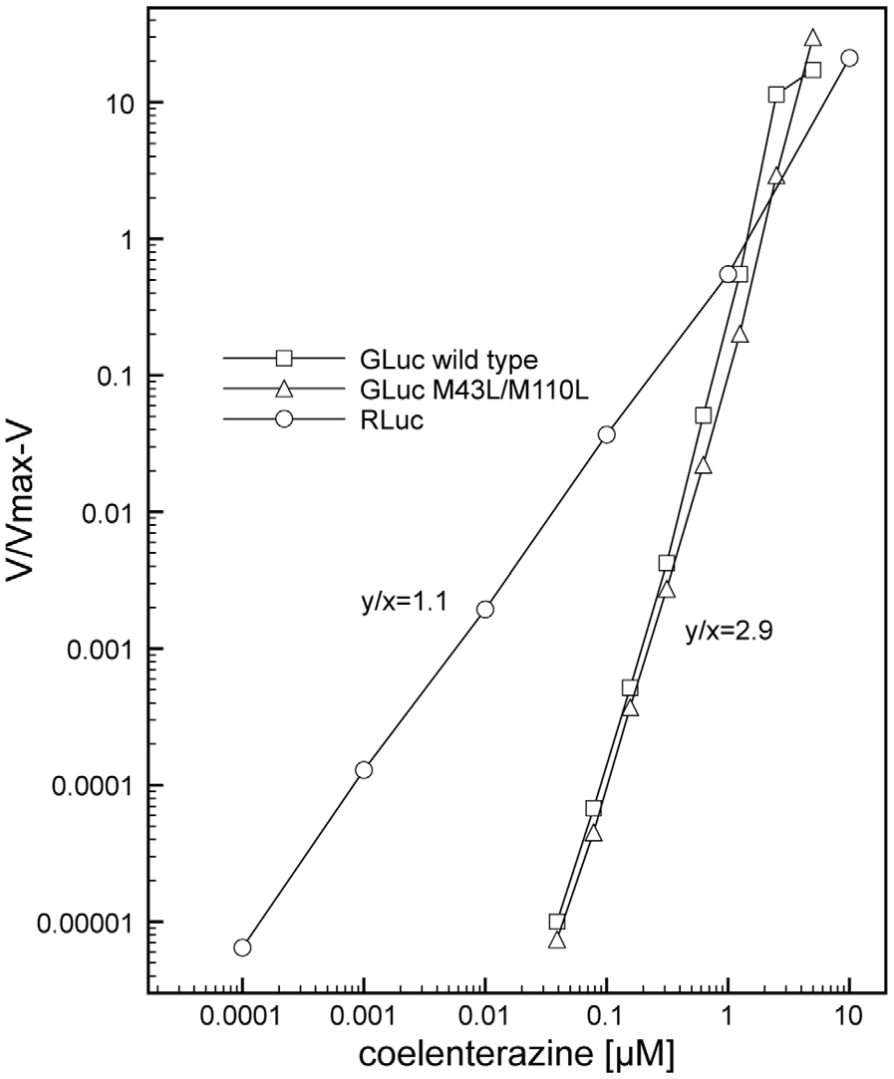
Hill plots of GLuc variants and RLuc. The Hill coefficient for RLuc of 1.1 and for the GLuc variants of 2.9 was determined by calculating the slope, y/x.

Cooperativity is expressed by the Hill plot shown in Figure 4 and demonstrates the positive cooperative response of the GLuc activity with respect to coelenterazine. The slope y/x = 2.9 for both GLuc variants indicates a degree of cooperativity requiring, at a minimum, two binding sites for coelenterazine. In contrast the slope of the Hill plot for RLuc rates, y/x = 1.1, indicates a non-cooperative reaction where binding a single molecule of coelenterazine is sufficient for catalysis [19].

## Discussion

**Figure 5:**
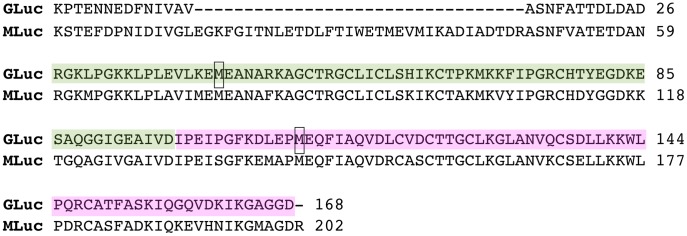
Alignment of the GLuc and MLuc Proteins. The GLuc half molecules described by Inouye and Sahara are shaded in green (amino terminal) and pink (carboxyl terminal). The GLuc methionine residues 43 and 110 are boxed.

We observe a substantial difference in the kinetic properties of two copepod luciferases (Gaussia and Metridia) from those of other marine luciferases (Renilla and Cypridina). These luciferases display positive cooperativity with regard to luciferin concentration, suggesting multiple substrate molecules must be bound for efficient catalysis. The Hill coefficient of greater than two reflects the phenomenon of cooperativity rather than an accurate estimate of the number of binding sites [20]. Inouye and Sahara [1] have identified the two repeat sequences corresponding to two catalytic domains in GLuc (Figure 5). The duplication of a homologous sequence domain observed in both MLuc and GLuc likely account for the two binding sites we infer for the effector/substrate. When either the amino terminal or the carboxyl terminal half of GLuc were expressed separately, both were found to be capable of catalyzing the luciferase reaction and thus binding and oxidizing coelenterazine. Although Inouye and Sahara [1] demonstrated measurable activity with the half molecules of GLuc, they found the N-terminal and C-terminal domains possessed only about 2% and 1%, respectively, of the full length GLuc specific activity. In the context of the full-length protein, if a single binding domain could catalyze the reaction at 1% of the maximal rate in a non-cooperative manner, then the slope (Figure 4) should have a second phase with a value of 1 as the rate approaches 1% of the maximal rate. This was not the case as the Hill plot shows a slope greater than 2, which persists over a 6-log range of V/Vmax-V. This suggests that the residual activity seen in the separate half molecules may be due to disruption of the mechanism responsible for the allosteric effect or some non-covalent interaction of the separate half molecules to themselves. The arrangement of the two halves of the molecule in the full-length protein must occlude catalysis with only a single substrate-binding event. If cooperativity requires interaction between the two halves of the protein then one would predict that the cooperativity would be absent for the individual halves of GLuc. Furthermore, Welsh et al [5] have characterized several mutants of GLuc that show differences in specific activity and decay of the luminescent signal from wild type GLuc. Interestingly, they observed the strongest effect on the decay of the luminescent signal when both M43L and M110L mutations are present simultaneously as opposed to the individual mutations. Therefore a synergistic effect between the two domains of the GLuc protein is demonstrated, since each of these two residues reside in a different half-domain consistent with the cooperative effect described here.

### Biological Role

Bioluminescent marine organisms have been studied for more than a century and it has been proposed that their bioluminescence is used for purposes such as eluding predators, attracting prey, and communication [21]. The unexpected result of a positive cooperative substrate effect for GLuc and MLuc raises the inevitable question of its biological significance for the organism. Gaussia and Metridia are closely related copepods that are presumed to use bioluminescence for defense [21]. The produced bioluminescence is instantaneous and occurs when enzyme and substrate are mixed and exuded into the water environment, thereby making it difficult for the predator to locate its prey. The nature of the cooperative biochemical reaction perhaps assures that the light flash is short-lived, as dispersion (dilution) into the water will rapidly reduce the length of time when light is generated. While this may be the role of the higher order reaction kinetics for the substrate, it conceivably may also be a means to assure that the copepod does not emanate light from its body. The organism achieves this in part by separating its luciferin from the luciferase as they are contained in separate compartments. Additionally, the higher order reaction kinetics would assure that if low levels of luciferin escape and leak across membranes it would not lead to inadvertent generation of light. Interestingly the luciferase of the dinoflagellate Gonyaulax is also a multi-catalytic domain protein the individual modules of which show catalytic activity [22]. The evolutionary history of such multi-catalytic domain enzymes and its significance remains to be established [23], [24].

In this study, we have established that Renilla and Cypridina luciferases should be more appropriate tools for applications requiring the detection of small amounts of substrate as in small molecule detection assays because RLuc and CLuc respond to their luciferin concentration in a linear non-cooperative manner. This results in a much lower threshold of detection of the luciferin. While GLuc and Mluc are perfectly suitable as reporter molecules, use for detection of low levels of coelenterazine is problematic, because of the cooperativity with respect to their luciferin. Furthermore cognizance of the cooperative nature of GLuc and MLuc should help the design and interpretation of whole animal imaging experiments. The three dimensional structure of GLuc remains to be elucidated and should shed significant light on the allosteric mechanism which achieves this remarkable cooperativity.

## Materials and Methods

### Reagents and Enzymes

Coelenterazine and cypridina luciferin were obtained from New England Biolabs (NEB).

The Metridia, Gaussia and Gaussia (M43L/M110L) variant were all purified from *E. coli* SHuffle cells (NEB) [25] harboring a plasmid encoding an amino terminal His-tagged luciferase gene controlled by T7 promoter/T7 RNA polymerase. The His-tagged Renilla luciferase was expressed in NEB T7 express cells. All amino acid sequences of the expressed luciferases are listed in Text S1. Crude extracts were obtained by sonication and clarified by centrifugation. The clarified extracts were then applied to nickel resin and the luciferases eluted with an imidazole gradient. The purified luciferases were dialyzed against 20 mM Tris-HCl pH 7.5, 200 mM NaCl, 50% glycerol and stored at −20°C. *Cypridina* luciferase was expressed from a stable CHO cell line constructed with the pCMV-CLuc-2 vector (NEB) (data not shown). The conditioned media containing the secreted luciferase was stored at −20°C and was used as the source for CLuc.

### Luciferase Assay

A Centro LB 960 luminometer (Berthold) was used to determine luciferase activity in relative light units (RLU). CLuc, RLuc, MLuc and GLuc luciferases were diluted in 10 mM Tris-HCl pH 7.5, 100 µg/mL BSA to a level where each luciferase resulted in 1–3×10^8^ RLU/10 s with 10 µM cognate luciferin (25 µM luciferin for MLuc). 50 µL of luciferase was injected into 50 µL of 10 mM Tris-HCl pH 7.5 containing various concentrations of luciferin. The RLUs were integrated over the first 10 seconds immediately after the enzyme was injected. Biolux GLuc Flex Assay Buffer was from NEB.

### Hill Plot

The initial velocity of the reactions at each concentration of substrate was inferred from the integration of the light units generated over the first ten seconds of the reaction. The Vmax value for each luciferase was determined at saturating substrate concentration.

## Supporting Information

Text S1 **Amino Acid Sequence of 5 Luciferases.**
(DOC)
