# Supplementary material for: Substrate Cooperativity in Marine Luciferases
Source: PLoS One. 2012 Jun 29;7(6):e40099. doi: 10.1371/journal.pone.0040099 (PMC3387026; doi:10.1371/journal.pone.0040099)
Supplement: Text S1 — Amino Acid Sequence of 5 Luciferases. (DOC) [file pone.0040099.s001.doc]

**Supporting Information:**

Amino acid sequence of 5 luciferases.

GLuc

MKIEEGKGHHHHHHGSKPTENNEDFNIVAVASNFATTDLDADRGKLPGKKLPLEVLKEMEANARKAGCTRGCLICLSHIKCTPKMKKFIPGRCHTYEGDKESAQGGIGEAIVDIPEIPGFKDLEPMEQFIAQVDLCVDCTTGCLKGLANVQCSDLLKKWLPQRCATFASKIQGQVDKIKGAGGDLE

GLuc M43L M110L

MKIEEGKGHHHHHHGSKPTENNEDFNIVAVASNFATTDLDADRGKLPGKKLPLEVLKELEANARKAGCTRGCLICLSHIKCTPKMKKFIPGRCHTYEGDKESAQGGIGEAIVDIPEIPGFKDLEPLEQFIAQVDLCVDCTTGCLKGLANVQCSDLLKKWLPQRCATFASKIQGQVDKIKGAGGDLE

MLuc

MKIEEGKGHHHHHHGKSTEFDPNIDIVGLEGKFGITNLETDLFTIWETMEVMIKADIADTDRASNFVATETDANRGKMPGKKLPLAVIMEMEANAFKAGCTRGCLICLSKIKCTAKMKVYIPGRCHDYGGDKKTGQAGIVGAIVDIPEISGFKEMAPMEQFIAQVDRCASCTTGCLKGLANVKCSELLKKWLPDRCASFADKIQKEVHNIKGMAGDR

RLuc

MKIEEGKGHHHHHHGSMTSKVYDPEQRKRMITGPQWWARCKQMNVLDSFINYYDSEKHAENAVIFLHGNAASSYLWRHVVPHIEPVARCIIPDLIGMGKSGKSGNGSYRLLDHYKYLTAWFELLNLPKKIIFVGHDWGACLAFHYSYEHQDKIKAIVHAESVVDVIESWDEWPDIEEDIALIKSEEGEKMVLENNFFVETMLPSKIMRKLEPEEFAAYLEPFKEKGEVRRPTLSWPREIPLVKGGKPDVVQIVRNYNAYLRASDDLPKMFIESDPGFFSNAIVEGAKKFPNTEFVKVKGLHFSQEDAPDEMGKYIKSFVERVLKNEQLETG

CLuc

MKTLILAVALVYCATVHCQDCPYEPDPPNTVPTSCEAKEGECIDSSCGTCTRDILSDGLCENKPGKTCCRMCQYVIECRVEAAGWFRTFYGKRFQFQEPGTYVLGQGTKGGDWKVSITLENLDGTKGAVLTKTRLEVAGDIIDIAQATENPITVNGGADPIIANPYTIGEVTIAVVEMPGFNITVIEFFKLIVIDILGGRSVRIAPDTANKGMISGLCGDLKMMEDTDFTSDPEQLAIQPKINQEFDGCPLYGNPDDVAYCKGLLEPYKDSCRNPINFYYYTISCAFARCMGGDERASHVLLDYRETCAAPETRGTCVLSGHTFYDTFDKARYQFQGPCKEILMAADCFWNTWDVKVSHRNVDSYTEVEKVRIRKQSTVVELIVDGKQILVGGEAVSVPYSSQNTSIYWQDGDILTTAILPEALVVKFNFKQLLVVHIRDPFDGKTCGICGNYNQDFSDDSFDAEGACDLTPNPPGCTEEQKPEAERLCNSLFAGQSDLDQKCNVCHKPDRVERCMYEYCLRGQQGFCDHAWEFKKECYIKHGDTLEVPDECK
